# Blood levels of T-Cell Receptor Excision Circles (TRECs) provide an index of exposure to traumatic stress in mice and humans

**DOI:** 10.1038/s41398-022-02159-7

**Published:** 2022-10-03

**Authors:** Kenneth M. McCullough, Seyma Katrinli, Jakob Hartmann, Adriana Lori, Claudia Klengel, Galen Missig, Torsten Klengel, Nicole A. Langford, Emily L. Newman, Kasey J. Anderson, Alicia K. Smith, F. Ivy Carroll, Kerry J. Ressler, William A. Carlezon

**Affiliations:** 1 Basic Neuroscience Division, Department of Psychiatry, Harvard Medical School, McLean Hospital, Belmont, MA USA; 2 Department of Gynecology and Obstetrics, Emory University, Atlanta, GA USA; 3 Department of Psychiatry & Behavioral Sciences, Emory University, Atlanta, GA USA; 4 Center for Organic and Medicinal Chemistry, Research Triangle Institute, Research Triangle Park, NC USA

**Keywords:** Physiology, Biomarkers

## Abstract

Exposure to stress triggers biological changes throughout the body. Accumulating evidence indicates that alterations in immune system function are associated with the development of stress-associated illnesses such as major depressive disorder and post-traumatic stress disorder, increasing interest in identifying immune markers that provide insight into mental health. Recombination events during T-cell receptor rearrangement and T-cell maturation in the thymus produce circular DNA fragments called T-cell receptor excision circles (TRECs) that can be utilized as indicators of thymic function and numbers of newly emigrating T-cells. Given data suggesting that stress affects thymus function, we examined whether blood levels of TRECs might serve as a quantitative peripheral index of cumulative stress exposure and its physiological correlates. We hypothesized that chronic stress exposure would compromise thymus function and produce corresponding decreases in levels of TRECs. In male mice, exposure to chronic social defeat stress (CSDS) produced thymic involution, adrenal hypertrophy, and decreased levels of TRECs in blood. Extending these studies to humans revealed robust inverse correlations between levels of circulating TRECs and childhood emotional and physical abuse. Cell-type specific analyses also revealed associations between TREC levels and blood cell composition, as well as cell-type specific methylation changes in CD4T + and CD8T + cells. Additionally, TREC levels correlated with epigenetic age acceleration, a common biomarker of stress exposure. Our findings demonstrate alignment between findings in mice and humans and suggest that blood-borne TRECs are a translationally-relevant biomarker that correlates with, and provides insight into, the cumulative physiological and immune-related impacts of stress exposure in mammals.

## Introduction

Exposure to severe or chronic stress is implicated in the etiology of major depressive disorder (MDD) and posttraumatic stress disorder (PTSD) [1]. These illnesses are increasingly prevalent and associated with pleiotropic effects, including dysregulation of mood, metabolism, and immune system function. The broad scope of these effects and their persistence across the lifespan highlights the need for noninvasive biomarkers that can provide insight into the quantity and physiological impact of previous exposures to stress.

T-cell receptor excision circles (TRECs) are circular DNA fragments produced by recombination events that occur during T-cell receptor (TCR) rearrangement and T-cell maturation in the thymus [2–6]. TRECs are a reliable indicator of new T-cells emigrating from the thymus [7] and have advantages as markers of thymic function, including the fact that they are easily detected and quantified in blood from both mice and humans via quantitative polymerase chain reaction (qPCR) [8–11]. Deficits in T-cell maturation (thymopoiesis) lead to alterations in the origin and characteristics of naive T-cells and potential changes in the ability to mount efficient immune responses. Such changes may contribute to general dysregulation of immune function and aberrant inflammatory responses, which have been directly implicated in vulnerability to conditions including MDD and PTSD as well as risk for suicide [12–16].

Thymic involution—a process involving death of thymic tissue, apoptosis of T-cell precursors (thymocytes), and decreased mass of the thymus gland—occurs naturally and progressively as organisms age, although it can be accelerated by physical and psychosocial stressors or exogenous administration of stress hormones or their analogues [17–19]. Glucocorticoids regulate diverse physiological reactions to stress, including dose-dependent thymic involution and adrenal hypertrophy [17, 20–23]. While the process of thymic involution can be quite rapid, the dynamics of T-cell turnover and proliferation create delays between the time of thymic injury and detection of decreases in T-cells and TRECs [20, 24]. Alterations in glucocorticoid signaling and hypothalamic-pituitary-adrenal (HPA) function have also been associated with behavioral, physiological, and molecular changes relevant to MDD and PTSD across species [25–27]. In humans, the physiological response to stress associated with childhood abuse and adult trauma is characterized, at least in part, by acute and lifelong immune dysregulation [28–33]. In particular, traumatic stress and PTSD are known to be associated with altered blood cell composition, immune cell prevalence, and accelerated immune system aging [34, 35]. DNA methylation (DNAm)-determined ‘age acceleration’ is a well-validated method for calculating the acceleration of the biological age of an organism with reference to chronological age. Acceleration of epigenetic age has been associated with decreased longevity and worse health outcomes, as well as prior exposure to traumatic stress and vulnerability to MDD and PTSD [36–42]. Advanced DNAm age is also strongly associated with immune dysregulation in the context of chronic stress [40, 43].

The present studies were designed to examine the translational relevance of TRECs as peripheral biomarker indicators of prior (historical) stress and its ongoing physiological manifestations. Proof-of-principle was first established in male mice using chronic social defeat stress (CSDS), an ethologically-relevant behavioral regimen that produces depressive- and anxiety-like phenotypes in mice including anhedonia, social avoidance, and sleep disturbances [44–48] that can be mitigated by antidepressants [49–51]. As part of the validation process, one cohort of mice was treated with a kappa-opioid receptor (KOR) antagonist (JDTic), a class of drugs previously shown to block stress effects [52]. Studies were then extended to men and women participants in the Grady Trauma Project (GTP)—a large study of the effects of stress in civilians—who had thoroughly-documented histories of exposure to trauma [27, 53]. Our findings suggest that decreased blood levels of TRECs, reflecting accelerated thymic involution, is a translationally relevant biomarker of cumulative stress exposure over the lifetime.

## Methods

### Mice

Male C57BL/6 J mice (8–10 weeks of age; Jackson Laboratories, Bar Harbor, ME) were used as target (traumatized) subjects and adult male CD1 mice (retired breeders 4-6 months of age; Charles River Laboratories, Wilmington, MA) were used as aggressors, as described [45]. Mice were housed in a temperature-controlled vivarium on a 12-hour light/dark cycle, with access to food and water except during testing. Procedures were approved by McLean Hospital Institutional Animal Care and Use Committee and conformed to National Institutes of Health guidelines.

### Chronic social defeat stress (CSDS)

CSDS was performed following a standardized protocol established by Golden and colleagues, as described [45, 47, 50, 53]. Behavioral experiments were performed during the light phase, beginning at the same time (09:00) each day and using the same sequence of mice. The CD1 mice were prescreened for aggression before the experiments, and they were used in a rotation so that no target (C57) mouse was exposed to the same aggressor (CD1) mouse more than once. The rotation included days where the CD1 mice were rested, followed by single session of rescreening [45]. Together, these approaches reduce the likelihood of non-aggressive encounters. CSDS was performed on 10 or 21 consecutive days. Mice in pilot studies received intraperitoneal (IP) pretreatment with the KOR antagonist JDTic (30 mg/kg) or saline vehicle, as described previously 24-h prior to the first defeat session [54]. Persistent attacks that could cause severe wounding were interrupted by the experimenter. Small (superficial) wounds were treated with triple antibiotic ointment to prevent infection. Mice with severe wounds—characterized by visible cuts or abrasions that caused skin flaps, persistent bleeding, or other wounds larger than ~0.5 cm that perforated the skin—or wounds of any size that persisted for multiple days or caused pain symptoms such as hunching and/or piloerection (beyond what is normal during these procedures) were removed from the experiment and euthanized. Likewise, mice that lost >20% of peak weight were removed from the analysis and euthanized. Twenty-four hours after final CSDS session, mice were euthanized; blood and tissues were collected for qPCR as described [47, 55], and adrenal and/or thymus glands were dissected and weighed.

### Quantitative polymerase chain reaction (qPCR)

For quantitative analyses, real-time (RT)-PCR was performed as described [8, 9] using an Applied Biosystems ViiA7 Real-Time PCR System. Primer sequences are listed in Supplemental Table I. Specific primers were used for the TREC signal joint in C57BL/6 J mice, and to compensate for input, the constant segment of C57BL/6J T-cell receptor alpha (TCRA) gene was measured as reference [8]. Similarly, specific primer sequences were used for the TREC signal joint in humans, and RNaseP was measured as reference [9]. Changes (delta, [d]) in cycle threshold (dCT) were calculated as CtTREC-CtTCRA for mice and CtTREC-CtRNaseP for humans, and only calculated for samples whose CtTREC was <35 with replicate standard deviation <0.5 Ct. For clarity in depicting the data, values are expressed as -dCT, with larger integer values indicating lower TREC levels. For the analyses in humans, case-control study design was not possible, preventing the use of fold-change calculations. The dCTs were normally distributed, enabling comparison of these values across conditions.

### Human samples

Individuals included in this study were participants in the GTP (Grady Trauma Project), which investigates the influence of genetic and environmental factors on responses to stressful life events in a predominantly African‐American urban population of low socioeconomic status [27]. Interviews were conducted in waiting rooms of primary care or obstetrical-gynecological clinics of a large, urban, public hospital in Atlanta, GA. Clinical and life experience information—including PTSD symptoms, trauma exposure and demographics—and blood samples were collected. The study was approved by the Institutional Review Board of Emory University School of Medicine and the Grady Health Systems Research Oversight Committee, and all participants provided written informed consent.

### Human measures

#### Traumatic events inventory (TEI)

The TEI was used to quantify exposure to traumatic events through the lifetime [27, 56]. This self-report instrument was developed for use with the GTP population [57, 58] and measures lifetime (childhood and adulthood) exposure to trauma such as natural disaster, serious accident or injury, and physical or sexual assault, as well as frequency of events, age at worst incident, and feelings of terror, horror, and helplessness. TEI score was operationalized as a continuous measure, ranging from 0 to 16.

#### Childhood trauma questionnaire (CTQ)

The CTQ was used to quantify childhood trauma. This self-report instrument assesses sexual, physical, emotional trauma and is based on established scores for mild, moderate and severe abuse for each type [59]. History of childhood trauma was dichotomized for each type of abuse (physical, sexual, and emotional) as not abused, indicating CTQ scores in the none to mild range, and abused, indicating CTQ scores in the moderate to severe range. Composite variable across physical, sexual, and emotional abuse was created. Using this composite variable, participants were categorized into 2 groups based on the presence of any type of moderate to severe abuse: (1) individuals with no type of abuse in the moderate to severe range were categorized as not exposed to childhood trauma, and (2) individuals with at least one type of abuse in the moderate to severe range were categorized as exposed to childhood trauma [60].

#### Beck depression inventory-II (BDI)

Depressive symptoms were assessed using the BDI, a 21-item questionnaire in which answers to each question are scored from 0 to 3, with higher scores indicating more severe depressive symptoms [60, 61].

#### PTSD scales

PTSD diagnosis was assessed by Clinician‐Administered PTSD Scale (CAPS) for DSM-IV [62, 63]. The CAPS is a structured diagnostic instrument for PTSD that has been shown to have excellent psychometric properties and provides a measure of lifetime and current PTSD [62, 63]. The Modified PTSD Symptomatic Scale (mPSS) is based on DSM-IV criteria and was used as a measure of current PTSD symptoms of intrusion, avoidance/numbing, and hyperarousal [64]. To be categorized in the PTSD group, subjects needed to report current symptoms falling within 3 symptom clusters: at least 1 intrusive symptom (B), 3 avoidance/numbing symptoms (C), and 2 hyperarousal symptoms (D) on the mPSS, with a duration of 1 month or greater (D) [65].

#### DNA methylation

DNA was extracted from whole blood using the EZNA Blood DNA Midi Kit (Omega Bio-tek, Norcross, GA) and examined using the Methylation EPIC BeadChip (Illumina) according to manufacturer’s instructions. Raw methylation beta values were determined via GenomeStudio (Illumina), with internal controls to assess the quality of staining, extension, hybridization, bisulfite conversion, and specificity. Samples with probe detection call rates <90% and those with an average intensity value of either <50% of the experiment-wide sample mean or <2,000 arbitrary units (AU) were removed using R package CpGassoc [66]. Probes with detection p-values >0.01 were set to missing, and CpG sites that cross hybridize between autosomes and sex chromosomes were removed [67]. A total of 819,380 probes passed QC and were used in subsequent analyses. Single-sample Noob (ssNoob) normalization method implemented in R package minfi was used for dye bias equalization [68]. Following normalization, the ComBat procedure in the R package SVA was used to remove chip and positional batch effects, controlling for age and PTSD status [69].

#### Leukocyte composition estimation

The proportions of CD8 + T, CD4 + T, NK, B-cells, monocytes (mono) and neutrophils were estimated using publicly available reference data (GSE110554) and Robust Partial Correlation (RPC) method implemented in R package Epidish [70]. Correlations between cell type compositions and TREC levels were computed using Pearson correlation test.

#### T-cell specific epigenome-wide association analysis (EWAS)

T-cell specific methylation differences associated with TREC levels were identified using TOols for the Analysis of heterogeneouS Tissues (TOAST) [71], which uses an interaction term to identify outcome-associated CpG sites for each cell type. Regression models controlled for age, sex, and smoking score and *P*-values were adjusted for multiple-testing using the Benjamini-Hochberg FDR procedure at 5% FDR level [72]. To explore the possible confounding effects of smoking, DNAm derived smoking scores were included as a covariate. Smoking score was generated by taking weights of an EWAS of smoking from DNA methylation data [73] and included effect sizes of 39 CpGs that were associated with smoking pack-years. Smoking scores were quantified by calculating product of the logit transformed beta values for these 39 CpG cites times the effect size estimates.

#### Epigenetic age acceleration measures

Epigenetic age measures were calculated using the new DNA methylation age calculator developed by Horvath [37, 74]. Age-adjusted versions of epigenetic age measures (i.e., Horvath DNAmAge acceleration, Hannum DNAmAge [HannumAge] acceleration, PhenoAge acceleration, GrimAge acceleration, intrinsic epigenetic age acceleration [IEAA], and extrinsic epigenetic age acceleration [EEAA]) were used in subsequent analyses.

### Statistics

For mouse experiments, analyses were performed using Prism 9 (Graphpad software, La Jolla CA). The Shapiro-Wilk test was used to assess the normality of the data, followed by t-tests, Mann-Whitney U tests, or analyses of variance (ANOVAs) with Holm–Sidak tests for *post-hoc* analyses. For experiments in humans, analyses were performed using R version 4.1.0. Associations between levels of TRECs with trauma and PTSD symptoms were tested using linear regression models adjusting for age and sex. Pearson correlation tests were used for all correlation analyses. The threshold for statistical significance was set at *p* < 0.05.

## Results

### Impacts of CSDS in mice on thymus, adrenal glands, and TRECs

Initial studies were designed to determine if our 10-day CSDS regimen, which causes persistent effects on behavior and gene expression in brain, would also produce peripheral effects on the adrenal glands and thymus. This work did not involve concurrent behavioral testing, enabling us to isolate the effects of CSDS alone without the potentially confounding effects of other stressors such as additional handling and exposure to novel environments or conspecifics. For this reason, we used an a priori design that enabled us to verify endpoints that we have established as being reliable in this model. As seen previously [47], CSDS triggered changes in stress-associated transcripts *Creb1* and *Pdyn* in the nucleus accumbens (Supplemental Fig. 1A), a brain area implicated in the etiology of depressive disorders [75]. This regimen also produced adrenal hypertrophy (F(1,28)=11.31, *p* = 0.002, Sidak’s multiple comparisons: Saline-treated mice, control vs. CSDS, *p* < 0.001) (Supplemental Fig. 1B) and thymic involution (F(1,28)=104.9, *p* < 0.001; Sidak’s multiple comparisons: Saline-treated mice, control vs. CSDS, *p* < 0.001) (Supplemental Fig. 1C), which precedes reductions in TRECs [20, 24]. Additional pilot studies indicate that thymic involution is statistically significant after 5 CSDS sessions (data not shown). Pretreatment with the long-lasting KOR antagonist JDTic, which we have previously shown mitigates CSDS effects on endpoints including sleep disruption [54, 55], blocked CSDS effects on adrenal hypertrophy (JDTic, control vs. CSDS, *p* = 0.99) but not thymic involution (JDTic, control vs. CSDS, *p* < 0.001) (Supplemental Fig. 1B, C). These data confirm that a well-characterized CSDS regimen that produces depressive-like behavioral effects also produces indices of pathophysiological change in the adrenal glands and thymus.

**Fig. 1:**
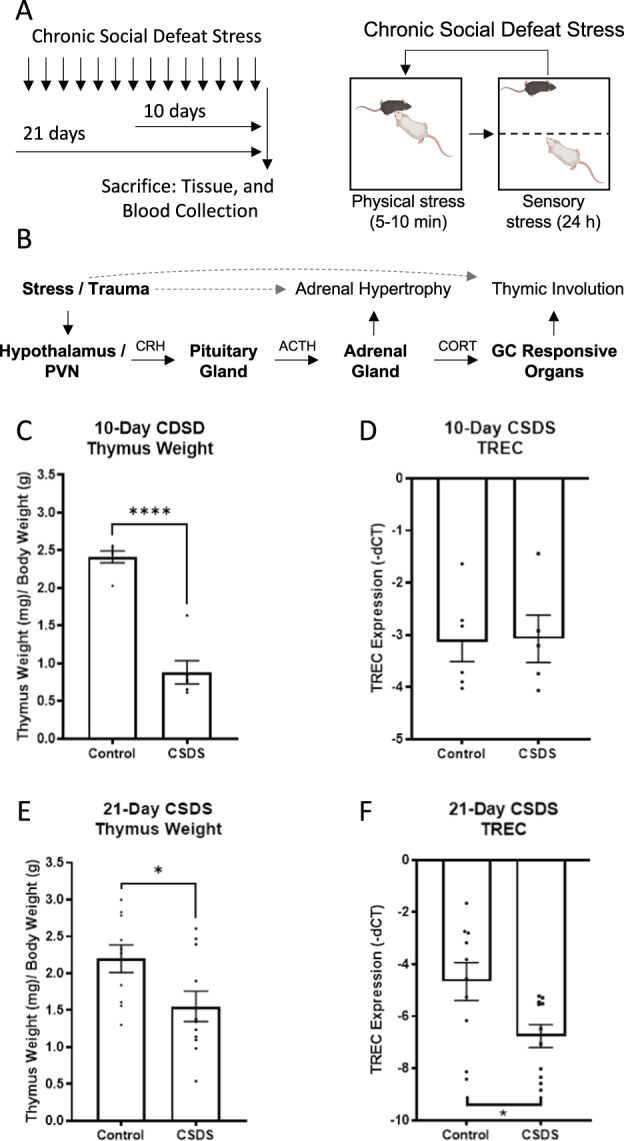
Chronic Social Defeat Stress (CSDS) effects on adrenal gland, thymus, and TREC levels in male mice. **A** Simplified schematic of the 10-day and 21-day CSDS regimens. **B** Simplified schematic of HPA axis activation and resulting adrenal hypertrophy and thymic involution. **C** The 10-day CSDS regimen led to thymic involution, indicated by decreased thymus weight, but **D** it did not affect TREC levels in whole blood. In contrast, **E** the 21-day CSDS regimen led to thymic involution and **F** decreased TREC levels. Note that TREC data are depicted as -dCT to clarify that higher dCT integer values indicate lower levels of TRECs. *p < 0.05, **p < 0.01, ****p < 0.0001, N’s = 6–11/group.

The next set of studies was designed to focus on evaluating the relationship among stress, thymic involution, and TREC levels in blood; for this work, we used 10-day and 21-day CSDS regimens to examine the latency at which any changes in blood levels of TRECs become detectable (Fig. 1A, B) [21, 76–78]. We again found that the 10-day CSDS regimen was sufficient to produce thymic involution (t[10] = 8.9, *p* < 0.0001) (Fig. 1C), but it did not alter blood levels of TRECs (t[10] = 0.1, *p* = 0.9) (Fig. 1D). For clarity, TREC levels are depicted as -dCT, such that larger integer values are lower on the y-axis, since larger CT values indicate lower expression levels (i.e., more PCR cycles needed for detection). In contrast, the 21-day CSDS regimen produced both thymic involution (t[19] = 2.3, *p* = 0.03) (Fig. 1E) and reductions in blood levels of TRECs (t[19] = 2.5, *p* = 0.02; alternatively, Mann-Whitney U test, *p* = 0.02) (Fig. 1F). TREC expression levels were also reduced when data are expressed as fold-change from control (ddCT) (Supplemental Fig. 1D, E). Considering that both regimens produced thymic involution, the differences between the 10-day and 21-day regimen likely reflect the minimum requisite time for the kinetics of T-cell loss and proliferation in the periphery to allow detection of reductions in blood levels of TRECs, which has been described previously [24]. These data provide proof-of-principle in mice that thymic involution and blood levels of TRECs are viable indicators of prior stress exposure in a well-controlled mammalian model.

### Associations between levels of TRECs with trauma and PTSD symptoms in humans

**Fig. 2:**
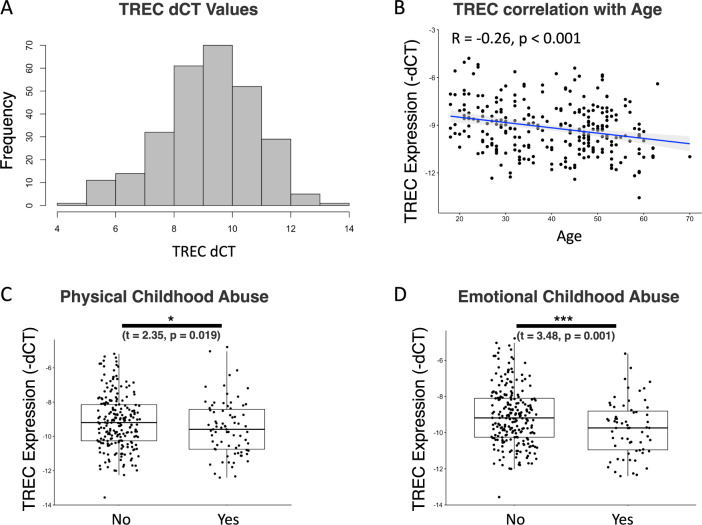
Associations between TREC levels and age and childhood trauma in humans (men and women). Note that TREC data are depicted as -dCT to clarify that higher dCT integer values indicate lower levels of TRECs. **A** TREC dCT values were normally distributed. **B** TREC levels were negatively correlated with age, and lower in subjects that experienced **C** childhood physical abuse or **D** childhood emotional abuse; for details, refer to Table 2. *p < 0.05, ** *p* < 0.01, ***p < 0.001, *N* = 297.

To examine associations between blood levels of TRECs and endpoints related to traumatic stress, we analyzed whole blood DNA samples from women and men participating in the GTP study. Participant demographic information used in the analyses is summarized in Table 1. TREC dCT values were normally distributed (Fig. 2A) and negatively correlated with age: older age was associated with lower TREC levels (R = −0.26, *p* < 0.001) (Fig. 2B). Consistent with findings in mice, regression models adjusting for age and sex demonstrated that reduced TREC expression level was robustly associated with certain types of prior histories of trauma (Table 2) including childhood physical abuse (t[292] = −2.3, *p* = 0.019) (Fig. 2C) and childhood emotional abuse (t[292] = −3.5, *p* = 0.001) (Fig. 2D).

**Table 1:** Demographic characteristics of the human cohorts.

Demographics	
Total samples	298
Age - mean (SD, range)	40.3 (12.38, 18–70)
Sex	Women = 176, Men = 122
Current PTSD using CAPS	Control = 206, Case = 61, NA = 31
Lifetime PTSD using CAPS	Control = 137, Case = 130, NA = 31
PSS avoidance	6.19 (5.85, 0-21), NA = 6
PSS intrusive	3.52 (3.91, 0-15), NA = 8
PSS hyperarousal	5.12 (4.36, 0-15), NA = 9
Childhood Abuse - Physical	No = 222, Yes = 75, NA = 1
Childhood Abuse - Sexual	No = 213, Yes = 84, NA = 1
Childhood Abuse - Emotional	No = 234, Yes = 63, NA = 1
TEI score	5.51 (3.29, 0-16), NA = 15
BDI score - mean (SD, range)	15.83 (11.94, 0-51), NA = 11

Demographic characteristics are described as counts for categorical variables and mean (SD, range) for continuous variables. *CAPS* Clinician-Administered PTSD Scale, *PSS* PTSD Symptom Severity, *BDI* Beck’s Depression Inventory, *NA* Not Available.

**Table 2:** Associations between TREC levels and stress-related metrics.

Association between TREC expression (-dCT) and stress measures				
	Estimate	SE	t-value	*p*-value
Current PTSD (CAPS)	−0.239	0.192	−1.25	0.213
Lifetime PTSD (CAPS)	−0.225	0.159	−1.412	0.159
PSS - Avoidance	−0.014	0.013	−1.067	0.287
PSS - Intrusive	−0.019	0.019	−0.997	0.319
PSS - Hyperarousal	−0.026	0.018	−1.46	0.146
Childhood Abuse - Physical	−0.396	0.168	−2.349	**0.019**
Childhood Abuse - Sexual	−0.146	0.17	−0.856	0.393
Childhood Abuse - Emotional	−0.617	0.177	−3.479	**0.001**
TEI Score	−0.036	0.023	−1.543	0.124
BDI Score	−0.009	0.007	−1.323	0.187

Statistically significant (bold) reductions in TREC levels during adulthood were associated with exposure to childhood physical abuse (*p* = 0.019) and childhood emotional trauma (*p* = 0.001). Results are from linear regression models adjusted for age and sex.

Because biological sex can influence responses to stress [79, 80], we also stratified our analyses across women (*N* = 176) and men (*N* = 122) (Supplemental Tables II and III). For both sexes, TREC dCT was normally distributed and TREC levels negatively correlated with age (Supplemental Figs. 2A, B and 3A, B). In women (Supplemental Table IV), TREC levels were associated with childhood physical abuse (t[173] = −2.6, *p* = 0.009) (Supplemental Fig. 2C), childhood emotional abuse (t[173] = −3.0, *p* = 0.003) (Supplemental Fig. 2D), Beck Depression Inventory (t[169] = −2.4, *p* = 0.02) (Supplemental Fig. 2E), and PSS hyperarousal score (t[172] = −2.3, *p* = 0.025) (Supplemental Fig. 2F). In men, these associations did not reach statistical significance (Supplemental Table V), which may be due to the smaller sample size or possible clinical differences in men and women within this sample. Additional sensitivity analyses adjusting for trauma burden via the lifetime traumatic events inventory (TEI) indicate that TREC levels significantly associate with childhood physical abuse in combined and women stratified analysis, as well as childhood emotional abuse across combined and sex-stratified (women and men) analyses (Supplemental Tables VI–VIII). Taking into account the lack of association between TREC and TEI (Table 2; Supplemental Tables V and VI) into account, these analyses suggest that the effect of childhood trauma exposure on TREC levels is independent of adulthood trauma. Overall, these data also support lower TREC levels in the blood related to greater trauma exposure, PTSD, and depression symptoms, with larger effects in women.

### Associations between levels of TRECs and cell composition

**Fig. 3:**
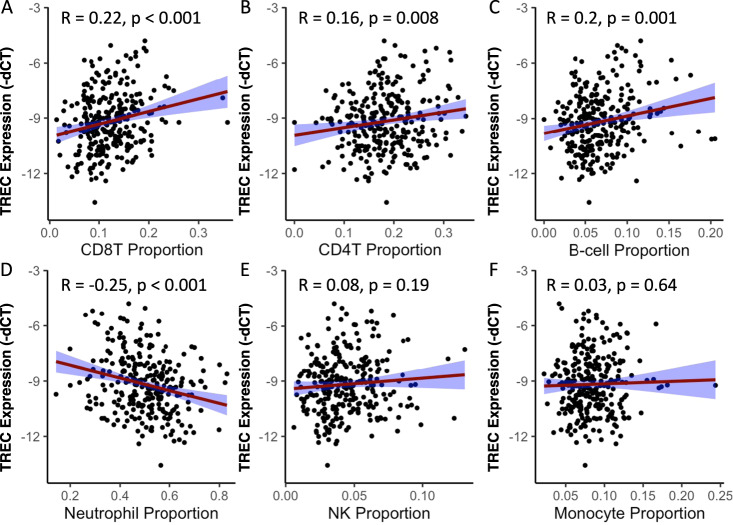
Associations between TREC levels and measures of leukocyte cell composition in humans (men and women). Note that TREC data are depicted as -dCT to clarify that higher dCT integer values indicate lower levels of TRECs. Levels of TRECs were positively correlated with proportions of **A** CD8T cells, **B** CD4T cells, and **C** B-cells, and **D** negatively correlated with proportions of neutrophils. TREC levels were not correlated with proportions of **E** NK cells or **F** monocytes. *N* = 276.

To evaluate the relationship between levels of TRECs and alterations in blood cell composition, epigenetic age acceleration, and DNA methylation patterns, we analyzed a subsample comprising 159 women and 117 men subjects, for which all of these endpoints were available. Consistent with the hypothesis that TREC levels are associated with alteration in the production and proliferation of immune cell populations, we found that this endpoint is significantly associated with changes in leukocyte cell composition. Specifically, lower levels of TRECs were associated with decreased proportions of CD8T cells (R = 0.22, *p* < 0.001) (Fig. 3A), CD4T cells (R = 0.16, *p* = 0.0079) (Fig. 3B), and B-cells (R = 0.22, *p* = 0.0011) (Fig. 3C), and increased proportion of neutrophils (R = -0.25, *p* < 0.001) (Fig. 3D). Levels of TRECs were not associated with the proportions of neutral killer (NK) cells (Fig. 3E) or monocytes (Fig. 3F). In general, these significant correlations between levels of TRECs and cell composition were maintained in sex-stratified analyses (Supplemental Figs. 4 and 5), although the significant correlation between TRECs and B-cell proportion was observed in men but not in women (Supplemental Figs. 4C and 5C). Overall, these data support that TREC levels associate with immune cell composition reflective of an inflammatory environment and lower TREC levels may indicate inflammation.

Since immune system dysfunction is often observed in stress-related disorders, we conducted additional analyses to evaluate the associations between stress-related metrics and leukocyte proportions (Supplemental Tables IX–XI). We observed that higher CD8T proportions associated with sexual childhood abuse (*p* = 0.008); lower CD4T proportions associated with physical (*p* = 0.02) and emotional childhood abuse (*p* = 0.01); and higher NK proportions associated with sexual childhood abuse (*p* = 0.04). In women, lower B cell composition was associated with physical childhood abuse (*p* = 0.04). In men, higher CD8T proportions associated with sexual childhood abuse (*p* = 0.001); lower CD4T proportions associated with physical (*p* = 0.02) and emotional childhood abuse (*p* = 0.01); lower NK proportions associated with higher PSS hyperarousal symptoms (*p* = 0.02); and increased monocyte proportions associated with higher BDI score (*p* = 0.03). These analyses support an association of childhood trauma with T-Cell proportions as indicated by findings in TREC levels.

### Associations between levels of TRECs and epigenetic age acceleration

**Fig. 4:**
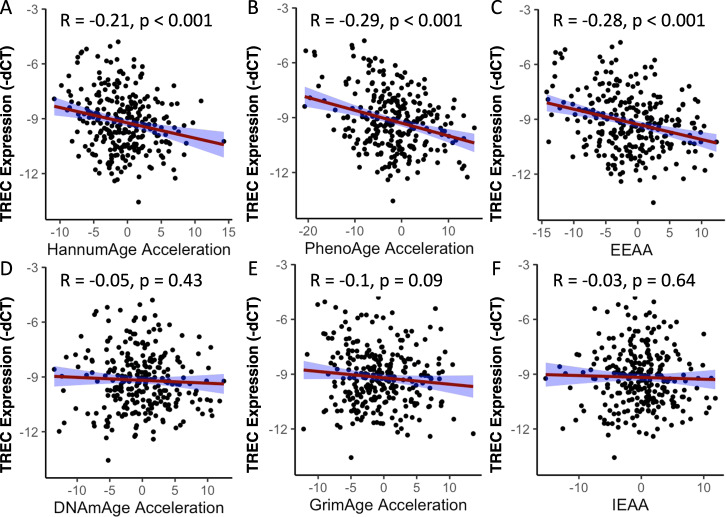
Associations between TREC levels and measures of epigenetic age acceleration in humans (men and women). Note that TREC data are depicted as -dCT to clarify that higher dCT integer values indicate lower levels of TRECs. Levels of TRECs were negatively correlated with **A** HannumAge acceleration, **B** PheonAge acceleration and **C** extrinsic epigenetic age acceleration (EEEA). TREC levels were not correlated with **D** DNAmAge acceleration, **E** GrimAge acceleration, or **F** intrinsic epigenetic age acceleration (IEAA). *N* = 276.

Stress and trauma are associated with accelerated epigenetic age across multiple psychiatric conditions, including MDD and PTSD [38]. To identify if diminished levels of TRECs are also associated with acceleration of epigenetic age in the overall sample, we tested correlations between TREC levels and six epigenetic age acceleration measures. Remarkably, TREC levels strongly negatively correlate with HannumAge acceleration (R = −0.21, *p* < 0.001) (Fig. 4A), PhenoAge acceleration (R = −0.29, *p* < 0.001) (Fig. 4B), and extrinsic epigenetic age acceleration (EEAA) (R = −0.28, *p* < 0.001) (Fig. 4C), all of which are endpoints that reflect immune system aging. Levels of TRECs did not correlate with DNAmAge acceleration (Fig. 4D), GrimAge acceleration (Fig. 4E), and intrinsic epigenetic age acceleration (IEAA) (Fig. 4F). Of these endpoints, DNAmAge acceleration and IEAA calculate DNAmAge acceleration independent from changes in blood composition that are characteristic of immune system aging. In general, these significant correlations between levels of TRECs and measures of accelerated methylation age were maintained in sex-stratified analyses (Supplemental Figs. 6, 7), although there was also a negative correlation between levels of TRECs and GrimAge acceleration being observed in men (Supplemental Fig. 7E). Overall, these data support the findings that decreased TREC levels may serve as an important biomarker of increased cumulative stress exposure and increased epigenetic measures of aging.

### Associations between levels of TRECs and DNA methylation

**Fig. 5:**
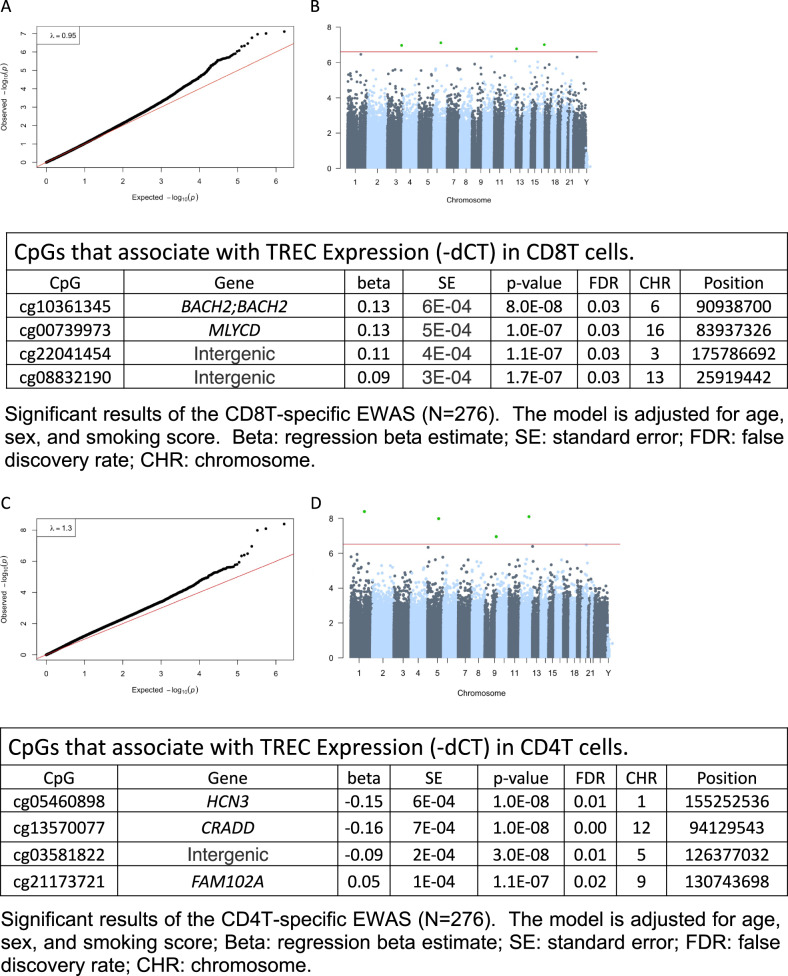
Associations between TREC levels and cell-type specific epigenome-wide association analysis (EWAS) in CD4T and CD8T cells in humans (men and women). **A** QQ plots of CD8T cell specific EWAS for the comparison of 276 samples for which both TREC expression levels and methylation data were available. **B** Manhattan plot of CD8T cell specific EWAS of TREC levels. The y-axis is the –log10 of the unadjusted *p*-value for the association with TREC expression level. The red line indicates genome-wide EWAS statistical significance at FDR < 0.05; for details, refer to Table 3. **C** QQ plots of CD4T cell specific EWAS for the comparison of 276 samples for which both TREC expression level and methylation data were available. **D** Manhattan plot of CD4T cell specific EWAS of TREC level. The y-axis is the –log10 of the unadjusted p-value for the association with TREC expression level. The red line indicates genome-wide EWAS statistical significance at FDR < 0.05; for details, refer to Table 4.

Because TRECs are only found in T-cells, we performed both overall and sex-stratified cell-type specific methylation analysis for CD4T and CD8T cells. We identified 9 CpG sites associated with levels of TRECs in CD8T cells, and 2 CpG sites in CD4T cells (FDR < 0.05) (Fig. 5). There was no overlap between significant sites observed in CD8T and CD4T cells. In women, TREC levels associated with 17 CpG sites in CD8T cells and 2 CpGs in CD4T cells (FDR < 0.05) (Supplemental Fig. 8, [Inset] Supplemental Tables XII and XIII). In men, TREC levels were associated with only 1 CpG cite in CD8T cells (FDR < 0.05) (Supplemental Fig. 9, [Inset] Supplemental Table XIV). EWAS data identifies cell-type specific methylation changes associated with TREC levels that are germane to cellular aging, immune function, and T cell function. These methylation changes may reflect effects of stress on cellular function and immune response signified by alterations in TREC levels. Overall, these data support that TREC levels associate with DNA methylation signatures specific to T-cells.

## Discussion

Here we establish that blood levels of TRECs are a robust and quantifiable biomarker for cumulative stress exposure and its physiological consequences. TRECs are an established proxy for thymic output, which is reduced following thymic involution and is reflected by decreases in new thymic emigrants (e.g., naive T-cells) [81, 82]. Thymic involution is known to occur in response to physical and psychosocial stress as well as in the presence of and in conjunction with elevated endogenous or exogenous glucocorticoids [76–78, 83]. Specifically, in mice, we found that stress exposure causes reductions in levels of TRECs, which are readily quantified in blood using standard PCR methodologies and cycle threshold analyses. The finding is translationally relevant, as it was first established in an ethological model for stress in mice (CSDS) and then extended to humans with documented histories of exposure to stress and trauma. Although the association was detectable in a sample comprising men and women, it was most pronounced in women, with a strong but non-significant trend in men despite a considerably smaller available sample size and lower reported levels of prior trauma and depression symptomatology (e.g., BDI scores; Supplemental Tables I and II). Furthermore, decreased TREC levels in humans is associated with stress-sensitive physiological endpoints including leukocyte composition, DNA methylation, and accelerated DNA methylation age in both sexes. Overall, in humans, the associations were stronger with the endpoints from the biological assays than the self-report instruments, consistent with the hypothesis that quantitative biomarkers may be better proxies for the biological effects of stress than subjective self-report instruments. By demonstrating that TRECs are a reliable indicator of historical stress in mice and humans, we have identified an easily quantifiable, inexpensive, and noninvasive biomarker that can provide insight on the quantity and physiological impact of stress exposure.

In male mice, both the 10- and 21-day CSDS regimen consistently led to both adrenal hypertrophy and thymic involution. Interestingly, however, only the 21-day regimen led to reductions in TREC levels in blood. This finding is consistent with previous reports focusing on the time course of T-cell population maintenance via new thymic emigrants, which indicate that it takes weeks to detect decreased T-cell abundance following thymectomy [24]. Future studies might clarify whether a 10-day CSDS regimen followed by 11 days of no treatment would produce the same effects on TREC levels as the 21-day CSDS regimen, but this detail would not alter the proof-of-principle that regimens of chronic stress in mice produce this blood-borne biomarker of a history of stress exposure. Although we did not examine the behavioral phenotypes resulting from CSDS in these studies—to ensure that stress associated with the behavioral testing procedures would not confound measurement of CSDS-associated physiological changes—we have previously shown that it produces long-lasting increases in the core features of depressive illness, including social avoidance, anhedonia, and sleep disruption [47, 50, 54]. In addition, these early studies were conducted only in male mice; while CSDS can produce similar outcomes in females [51], it often requires the use of specialized methodologies that have not yet been shown to produce effects on a full range translationally relevant endpoints, making it difficult to perform these studies in both sexes while using an experimental design that does not enable corresponding behavioral analyses. Regardless, the fact that these studies in male mice predicted effects seen in analyses comprising women and men support the translational relevance of CSDS as a model useful for studying the effects of traumatic stress. Additionally, the ability of KOR antagonists—which can mitigate the long-lasting effects of stress on a variety of endpoints including sleep [52, 54]—to block CSDS effects on adrenal atrophy but not thymic involution provides insights on the mechanisms by which this class of drugs produce their putative therapeutic effects. One possibility is that KOR antagonists do not block chronic stress effects on immune function; indeed, if anything, KOR antagonists appear to increase pro-inflammatory factors following immune system activation [84].

Considering the complex interplay between thymic output and peripheral proliferation of T-cells in humans, measuring TREC levels does not directly indicate reductions in naïve T-cells [24]. However, we demonstrate associations between TRECs and cell composition—especially decreases in CD4T and CD8T cell proportions—that suggest important relationships among stress, thymic function, and leukocyte level. Furthermore, our findings are consistent with literature demonstrating thymectomy in children alters cell composition, immune activity, and causes premature immune aging [11, 85]. Together, these findings might provide early insight into why the correlations between TREC levels and childhood trauma (emotional and physical abuse) are particularly strong.

Levels of TRECs also correlate with numerous indicators of accelerated epigenetic aging. Hannum DNAmAge, which is optimized for blood samples, indicates that loss of TRECs is associated with accelerated methylation age, whereas Horvath DNAmAge, which is optimized across multiple tissue types, did not reveal this type of association. Furthermore, EEAA (Extrinsic epigenetic age acceleration)—which takes into account age-associated blood cell composition changes and immune system aging—significantly associates with TREC levels. Conversely, IEAA (Intrinsic epigenetic age acceleration) is not associated with TREC levels—most likely because it does not account for changes in age-associated cell composition. One potential explanation for these findings is that the epigenetic aging metrics that are optimized for blood samples, the sample type utilized here, and blood-related immune aging (e.g., Hannum and EEAA) provide the most robust signal between TREC and accelerated aging. This suggests that different tissues may acquire different age associated methylation signatures and that TRECs, which particularly represent thymic and blood-based immune measures, are most well represented by aging calculators optimized for these variables.

The EWAS (epigenome-wide analyses) of differentially methylated genes associated with peripheral TREC levels identified numerous genes associated with immune function, T-cell regulation, and immune-stress interactions. In the combined analyses, TREC levels were associated with DNAm in several well-characterized genes in CD8T and CD4T cells. Many of the identified differentially methylated genes have important roles in T-cell and immune function. As an example, *BACH2* is a transcription factor that regulates lymphocyte development [86], and polymorphisms in *BACH2* have been associated with some autoimmune conditions [87]. It has been reported that *BACH2* participates in oxidative stress-mediated apoptosis and is involved in innate immunity and adaptive immune responses [88]. *MLYCD* is a gene critical for fatty acid metabolism, energy homeostasis, and regulator of cellular and immune function [89]. *CRADD* has both been implicated as a regulator of T-cell activation and survival as well as immune responses [90, 91]. In the male-only analysis (Supplemental Table VII), the CD8T cells differentially methylated *RAP1GAP2* gene has been associated with asthma [92], an autoimmune, stress-related syndrome. When the analyses included women only, TREC levels were associated with additional genes. This finding has intriguing implications: for instance, *SPATA2* has been linked to TNFa receptor signaling [93], and *TTRAP* was associated with TNF and NFKB signaling [94], suggesting that TREC levels are related to epigenetic regulation of the innate immune response. Finally, *SLC25A24* regulates energy metabolism and has been associated with cellular aging and cortical gray matter volume [95]. Together these findings further suggest that blood levels of TRECs are associated with multiple epigenetic pathways related to immune function, T-cell regulation, and immune-stress interactions in humans.

We acknowledge that these early studies have several important limitations. While laboratory animals such as mice can be used to study critical aspects of stress-related conditions such as PTSD [96], they remain imperfect models for complex psychiatric disorders in humans. In the present studies, although both the mice and human subjects were evaluated for TREC levels during adulthood, the mice were exposed to stress (CSDS) only during adulthood whereas the data from the human subjects reflect the combined effects of childhood plus adult trauma and stress. While future studies in mice should explore stress effects and thymic size over a more comprehensive range of ages, our findings nonetheless provide proof-of-principal that TREC levels can serve as a marker of accumulated stress over time in mammals. In fact, the finding that quantitative measurement of TREC levels in blood is sensitive to stressors that occur at any time during the lifespan—instead of, for example, only early in life—can be perceived as a strength rather than a limitation of this putative biomarker. It is also important to note that our data are correlational and not causal, and that future studies are needed to more fully understand the mechanisms by which stress-dependent decreases in TREC levels occur, whether changes in TREC levels affect inflammatory processes or brain function, and the time course of stress-related changes in TREC levels. While a goal of these initial studies was to explore associations among blood levels of TRECs, stress exposure, and markers of immune function and premature stress-related aging across species, future work is needed to further examine the roles of factors including sex differences, cortisol, circadian rhythms, and adrenal makers, and to determine whether TREC levels represent a transformative biomarker that could be used to provide objective insight into stress susceptibility and resilience. Finally, our data do not yet quantify the relative importance of stress intensity and stress chronicity in determining the magnitude of changes in TREC levels; considering the human data that more robust TREC-related associations are seen with childhood trauma exposure than adult symptom reports, we speculate that chronicity may be a more important driver. However, it is exceedingly difficult to disaggregate the effects of repeated trauma over time in a multiply-traumatized human population, placing this question outside the scope of this initial report.

The fact that stress exacerbates the effects of aging on the immune system resulting in immune system dysfunction, together with the demonstration that TREC levels are a sensitive measure of accelerated immune system aging, suggests that TREC levels in blood may represent a quantitative biomarker of cumulative stress exposure. These new findings confirm and extend previous reports in humans that epochs of increased stress (e.g., economic hardship) can affect thymic aging and TREC levels [97]. While our findings do not rule out the role of TRECs in representing other physiological signals (e.g., aging, medical conditions, etc.), this observation provides a novel, translationally-relevant method with which to quantify stress exposure across species. The availability of this type of biomarker has many potential applications in psychiatry. Foremost, it may serve as an objective measure that facilitates the diagnosis of stress-related conditions such as PTSD. It might also be helpful in assessing risk of future development of stress-related disorders, enabling the use of strategies to mitigate further exposures that can trigger or exacerbate these conditions. Finally, it represents a tool that could be used to assess the ability of new therapeutics to slow or even reverse the biological processes that reduce levels of circulating TRECs, which may be useful in the treatment or prevention of currently intractable stress-related disorders.

## Supplementary information

Supplemental Materials
