# Supplementary material for: Blood levels of T-Cell Receptor Excision Circles (TRECs) provide an index of exposure to traumatic stress in mice and humans
Source: Transl Psychiatry. 2022 Oct 3;12:423. doi: 10.1038/s41398-022-02159-7 (PMC9530209; doi:10.1038/s41398-022-02159-7)
Supplement: Supplementary file 1 — Supplemental Materials [file 41398_2022_2159_MOESM1_ESM.docx]

For Submission to: *Molecular Psychiatry*

**Blood levels of T-Cell Receptor Excision Circles (TRECs) provide an index of exposure to traumatic stress in mice and humans**

Kenneth M. McCullough^1*^, Seyma Katrinli^2*^, Jakob Hartmann^1^, Adriana Lori^3^, Claudia Klengel^1^, Galen Missig^1^, Torsten Klengel^1^, Nicole A. Langford^3^, Emily Newman^1^, Kasey Anderson^1^, Alicia K. Smith^2,3^, F. Ivy Carroll^4^, Kerry J. Ressler^1,3^, and

William A. Carlezon Jr.^1^

^1^Basic Neuroscience Division, Department of Psychiatry, Harvard Medical School, McLean Hospital, Belmont, MA, USA.

^2^Department of Gynecology and Obstetrics, Emory University, Atlanta, GA, USA.

^3^Department of Psychiatry & Behavioral Sciences, Emory University, Atlanta, GA, USA.

^4^Center for Organic and Medicinal Chemistry, Research Triangle Institute, Research Triangle Park, NC USA

*These authors contributed equally to this work

**Abbreviated title:** Blood-borne TRECs as biomarkers of traumatic stress

**SUPPLEMENTAL MATERIALS**

**SUPPLEMENTAL TABLES**

**Supplemental Table I. Primer Sequences utilized for TREC RT-PCR**

| **Primer Name** | **Sequence** |
| --- | --- |
| mTREC C57 Forward | 5’-CCAAGCTGACGGCAGGTTT-3’ |
| mTREC C57 Reverse | 5’-AGCATGGCAAGCAGCACC-3’ |
| mTREC C57 Probe | 5’-FAM/TGCTGTGTG/ZEN/CCCTGCCCTGCC/3IABkFQ-3’ |
| mTCRA C57 Forward | 5’-TGACTCCCAAATCAATGTG-3’ |
| mTCRA C57 Reverse | 5’-GCAGGTGAAGCTTGTCTG-3’ |
| mTCRA C57 Probe | 5’-FAM/TGCTGGACA/ZEN/TGAAAGCTATGGA/3IABkFQ-3’ |
| hTREC Forward | 5’-CACATCCCTTTCAACCATGCT-3’ |
| hTREC Reverse | 5’-GCCAGCTGCAGGGTTTAGG3’ |
| hTREC Probe | 5’FAM/ACACCTCTG/ZEN/GTTTTTGTAAAGGTGCCCACT-/3IABkFQ-3’ |
| hRNaseP Forward | 5’-CGTTCTCTGGGAACTCACCT-3’ |
| hRNaseP Reverse | 5’-GTCACTCCACTCCCATGTCC-3’ |
| hRNaseP Probe | 5’-FAM/CCTCTGGCC-/ZEN/CTAGTCTCAGA/3IABkFQ-3’ |

**Supplemental Table II**. Demographic characteristics of GTP women in sex-stratified analyses.

| **Demographics, Women Samples** | |
| --- | --- |
| Total samples | 176 |
| Age - mean (SD, range) | 37.71 (12.31, 18-63) |
| Current PTSD using CAPS | Control=109, Case=47, NA=20 |
| Lifetime PTSD using CAPS | Control=70, Case=86, NA=20 |
| PSS avoidance | 6.8 (6.29, 0-21) |
| PSS intrusive | 3.99 (4.08, 0-15) |
| PSS hyperarousal | 5.84 (4.47, 0-15), NA=1 |
| Childhood Abuse - Physical | No=132, Yes=44 |
| Childhood Abuse – Sexual | No=108, Yes=68 |
| Childhood Abuse - Emotional | No=135, Yes=41 |
| TEI score | 5.34 (3.86, 0-16) |
| BDI score – mean (SD, range) | 18.51 (12.1, 0-48), NA=4 |

**Supplemental Table III**. Demographic characteristics of GTP men in sex-stratified analysis.

| **Demographics, Men Samples** | |
| --- | --- |
| Total samples | 122 |
| Age – mean (SD, range) | 44.06 (11.54, 19-70) |
| Current PTSD using CAPS | Control=97, Case=14, NA=11 |
| Lifetime PTSD using CAPS | Control=67, Case=44, NA=11 |
| PSS avoidance | 5.26 (4.98, 0-20), NA=6 |
| PSS intrusive | 2.78 (3.53, 0-14), NA=8 |
| PSS hyperarousal | 4.02 (3.98, 0-13), NA=8 |
| Childhood Abuse – Physical | No=90, Yes=31, NA=1 |
| Childhood Abuse – Sexual | No=105, Yes=16, NA=1 |
| Childhood Abuse – Emotional | No=99, Yes=22, NA=1 |
| TEI score | 5.8 (3.14, 0-14), NA=15 |
| BDI score - mean (SD, range) | 11.81 (10.52, 0-51), NA=7 |

**Supplemental Table IV**. Associations between TREC levels and stress measures in women.

| **Association between TREC expression levels (-dCT) and stress measures in Women** | | | | |
| --- | --- | --- | --- | --- |
|  | Estimate | SE | t-value | p-value |
| Current PTSD (CAPS) | -0.262 | 0.199 | -1.314 | 0.191 |
| Lifetime PTSD (CAPS) | -0.156 | 0.184 | -0.852 | 0.395 |
| PSS – Avoidance | -0.016 | 0.014 | -1.166 | 0.245 |
| PSS – Intrusive | -0.03 | 0.021 | -1.391 | 0.166 |
| PSS - Hyperarousal | -0.043 | 0.019 | -2.26 | **0.025** |
| Childhood Abuse - Physical | -0.515 | 0.196 | -2.632 | **0.009** |
| Childhood Abuse - Sexual | -0.107 | 0.177 | -0.605 | 0.546 |
| Childhood Abuse - Emotional | -0.61 | 0.199 | -3.061 | **0.003** |
| TEI score | -0.045 | 0.026 | -1.766 | 0.079 |
| BDI Score | -0.017 | 0.007 | -2.339 | **0.02** |

Statistically significant (**bold**) associations between TREC levels and stress measures in women. The results are from linear regression models adjusted for age.

**Supplemental Table V.** Associations between TREC expression levels and stress measures in men.

| Association between TREC Expression (-dCT) and Stress Measures in Men | | | | |
| --- | --- | --- | --- | --- |
|  | Estimate | SE | t-value | p-value |
| Current PTSD (CAPS) | 0.15 | 0.419 | -0.358 | 0.721 |
| Lifetime PTSD (CAPS) | 0.305 | 0.285 | -1.07 | 0.287 |
| PSS - Avoidance | 0.007 | 0.027 | -0.246 | 0.806 |
| PSS – Intrusive | -0.004 | 0.039 | 0.099 | 0.921 |
| PSS - Hyperarousal | -0.008 | 0.035 | 0.24 | 0.811 |
| Childhood Abuse - Physical | 0.222 | 0.3 | -0.74 | 0.46 |
| Childhood Abuse - Sexual | 0.281 | 0.386 | -0.728 | 0.468 |
| Childhood Abuse - Emotional | 0.66 | 0.336 | -1.964 | 0.052 |
| TEI score | -0.018 | 0.046 | -0.387 | 0.700 |
| BDI Score | -0.008 | 0.013 | 0.591 | 0.556 |

Statistically significant (**bold**) associations between TREC levels and stress measures in men. The results are from linear regression models adjusted for age.

**Supplemental Table VI**. Associations between TREC levels and stress-related metrics controlled for lifetime trauma exposure

| **Association between TREC Expression (-dCT) and Stress Measures** | | | | |
| --- | --- | --- | --- | --- |
|  | Estimate | SE | t-value | p-value |
| Current PTSD (CAPS) | -0.139 | 0.206 | -0.674 | 0.501 |
| Lifetime PTSD (CAPS) | -0.131 | 0.174 | -0.755 | 0.451 |
| PSS - Avoidance | -0.006 | 0.014 | -0.427 | 0.670 |
| PSS - Intrusive | -0.002 | 0.022 | -0.099 | 0.921 |
| PSS - Hyperarousal | -0.012 | 0.020 | -0.591 | 0.555 |
| Childhood Abuse - Physical | -0.402 | 0.187 | -2.148 | **0.033** |
| Childhood Abuse - Sexual | -0.041 | 0.186 | -0.219 | 0.827 |
| Childhood Abuse - Emotional | -0.635 | 0.199 | -3.184 | **0.002** |
| BDI Score | -0.007 | 0.007 | -0.969 | 0.333 |

Statistically significant (**bold**) reductions in TREC levels during adulthood were associated with exposure to childhood physical abuse (*p*=0.033) and childhood emotional trauma (*p*=0.002). The results are from linear regression models adjusted for age, sex, and trauma exposure.

**Supplemental Table VII**. Associations between TREC levels and stress-related metrics controlled for lifetime trauma exposure in women

| **Association between TREC Expression (-dCT) and Stress Measures** | | | | |
| --- | --- | --- | --- | --- |
|  | Estimate | SE | t-value | p-value |
| Current PTSD (CAPS) | -0.171 | 0.218 | -0.787 | 0.433 |
| Lifetime PTSD (CAPS) | -0.061 | 0.199 | -0.309 | 0.758 |
| PSS - Avoidance | -0.006 | 0.015 | -0.412 | 0.681 |
| PSS - Intrusive | -0.016 | 0.024 | -0.673 | 0.502 |
| PSS - Hyperarousal | -0.033 | 0.022 | -1.493 | 0.137 |
| Childhood Abuse - Physical | -0.447 | 0.211 | -2.120 | **0.035** |
| Childhood Abuse - Sexual | 0.029 | 0.195 | 0.149 | 0.882 |
| Childhood Abuse - Emotional | -0.562 | 0.223 | -2.519 | **0.013** |
| BDI Score | -0.014 | 0.008 | -1.860 | 0.065 |

Statistically significant (**bold**) reductions in TREC levels during adulthood were associated with exposure to childhood physical abuse (*p*=0.035) and childhood emotional trauma (*p*=0.013). The results are from linear regression models adjusted for age and trauma exposure.

**Supplemental Table VIII.** Associations between TREC expression levels and stress measures controlled for lifetime trauma exposure in men.

| Association between TREC Expression (-dCT) and Stress Measures in Men | | | | |
| --- | --- | --- | --- | --- |
|  | Estimate | SE | t-value | p-value |
| Current PTSD (CAPS) | -0.038 | 0.452 | -0.084 | 0.933 |
| Lifetime PTSD (CAPS) | -0.230 | 0.325 | -0.705 | 0.482 |
| PSS - Avoidance | -0.004 | 0.032 | -0.125 | 0.901 |
| PSS – Intrusive | 0.027 | 0.044 | 0.613 | 0.541 |
| PSS - Hyperarousal | 0.030 | 0.041 | 0.727 | 0.469 |
| Childhood Abuse - Physical | -0.327 | 0.358 | -0.912 | 0.364 |
| Childhood Abuse - Sexual | -0.212 | 0.424 | -0.500 | 0.618 |
| Childhood Abuse - Emotional | -0.780 | 0.390 | -1.998 | **0.048** |
| BDI Score | 0.009 | 0.014 | 0.672 | 0.503 |

Statistically significant (**bold**) reductions in TREC levels during adulthood were associated with exposure to childhood emotional trauma (*p*=0.048). The results are from linear regression models adjusted for age and trauma exposure.

**Supplemental Table IX**. Associations between stress-related metrics and leukocyte proportions

|  | CD8T | CD4T | NK | Bcell | Mono | Neu |
| --- | --- | --- | --- | --- | --- | --- |
| Current PTSD (CAPS) | t=1.26  p=0.21 | t=-1.01  p=0.31 | t=0.32  p=0.75 | t=-0.81  p=0.42 | t=-0.67  p=0.50 | t=0.33  p=0.74 |
| Lifetime PTSD (CAPS) | t=1.27  p=0.20 | t=-0.86  p=0.39 | t=-0.41  p=0.69 | t=-0.26  p=0.79 | t=0.63  p=0.53 | t=-0.09  p=0.92 |
| PSS - Avoidance | t=1.16  p=0.25 | t=-0.22  p=0.82 | t=0.34  p=0.74 | t=-0.39  p=0.97 | t=1.36  p=0.18 | t=-0.74  p=0.46 |
| PSS - Intrusive | t=-0.29  p=0.77 | t=-1.61  p=0.11 | t=-0.71  p=0.48 | t=-1.27  p=0.20 | t=0.52  p=0.60 | t=1.31  p=0.19 |
| PSS - Hyperarousal | t=0.52  p=0.61 | t=0.02  p=0.99 | t=-1.64  p=0.10 | t=0.73  p=0.46 | t=0.23  p=0.81 | t=-0.21  p=0.84 |
| Childhood Abuse - Physical | t=0.18  p=0.86 | **t=-2.42**  **p=0.02** | t=-0.09  p=0.93 | t=-1.64  p=0.10 | t=0.13  p=0.89 | t=1.62  p=0.11 |
| Childhood Abuse - Sexual | **t=2.66**  **p=0.008** | t=-0.006  p=1.00 | **t=2.04**  **p=0.04** | t=-0.48  p 0.63 | t=0.61  p=0.55 | t=-1.47  p=0.14 |
| Childhood Abuse - Emotional | t=-0.01  p=0.99 | **t=-2.61**  **p=0.01** | t=-0.35  p=0.72 | t=-1.29  p=0.20 | t=0.70  p=0.48 | t=1.61  p=0.11 |
| TEI score | t=0.91  p=0.36 | t=-0.57  p=0.57 | t=-0.91  p=0.37 | t=-0.01  p=0.99 | t=-0.47  p=0.64 | t=0.20  p=0.84 |
| BDI Score | t=0.62  p=0.53 | t=0.21  p=0.83 | t=-0.45  p=0.65 | t=-0.23  p=0.81 | t=0.94  p=0.35 | t=-0.44  p=0.66 |

Statistically significant (**bold**) associations between stress-related metrics and leukocyte proportions, adjusted for age and sex.

**Supplemental Table X**. Associations between stress-related metrics and leukocyte proportions in women

|  | CD8T | CD4T | NK | Bcell | Mono | Neu |
| --- | --- | --- | --- | --- | --- | --- |
| Current PTSD (CAPS) | t = 1.91  p = 0.06 | t = -0.84  p = 0.40 | t = 1.23  p = 0.22 | t = -0.56  p = 0.58 | t = -0.80  p = 0.43 | t = -0.17  p = 0.86 |
| Lifetime PTSD (CAPS) | t = 1.23  p = 0.20 | t = -0.12  p = 0.90 | t = 0.91  p = 0.36 | t = 0.75  p = 0.45 | t = -0.48  p = 0.63 | t = -0.71  p = 0.48 |
| PSS - Avoidance | t = 0.49  p = 0.62 | t = -0.57  p = 0.57 | t = 1.08  p = 0.28 | t = -0.18  p = 0.85 | t = 1.09  p = 0.28 | t = -0.28  p = 0.78 |
| PSS - Intrusive | t = -1.59  p = 0.11 | t = -1.39  p = 0.17 | t = -0.08  p = 0.94 | t = -0.88  p = 0.38 | t = 0.67  p = 0.51 | t = 1.46  p = 0.15 |
| PSS - Hyperarousal | t = 0.55  p = 0.58 | t = -0.98  p = 0.33 | t = -0.37  p = 0.71 | t = 0.50  p = 0.61 | t = 0.02  p = 0.99 | t = 0.21  p = 0.83 |
| Childhood Abuse - Physical | t = -0.61  p = 0.054 | t = -1.16  p = 0.25 | t = 0.53  p = 0.60 | **t = -2.03**  **p = 0.04** | t = 0.16  p = 0.87 | t = 1.29  p = 0.20 |
| Childhood Abuse - Sexual | t = 1.12  p = 0.27 | t = 0.69  p = 0.49 | t = 1.49  p = 0.14 | t = -0.53  p = 0.59 | t = 0.09  p = 0.93 | t = -0.92  p = 0.36 |
| Childhood Abuse - Emotional | t = -0.26  p = 0.79 | t = -1.25  p = 0.21 | t = 0.04  p = 0.97 | t = -1.41  p = 0.16 | t = -0.52  p = 0.61 | t = 1.27  p = 0.21 |
| TEI score | t = 1.68  p = 0.10 | t = -1.09  p = 0.28 | t = 0.12  p = 0.90 | t = -0.38  p = 0.71 | t = 0.30  p = 0.76 | t = -0.07  p = 0.95 |
| BDI Score | t = -0.32  p = 0.75 | t = -0.29  p = 0.77 | t = -0.22  p = 0.82 | t = -0.60  p = 0.55 | t = -0.58  p = 0.56 | t = 0.62  p = 0.54 |

Statistically significant (**bold**) associations between stress-related metrics and leukocyte proportions, adjusted for age.

**Supplemental Table XI**. Associations between stress-related metrics and leukocyte proportions in men

|  | CD8T | CD4T | NK | Bcell | Mono | Neu |
| --- | --- | --- | --- | --- | --- | --- |
| Current PTSD (CAPS) | t=-0.47  p=0.64 | t=-0.55  p=0.59 | t=-1.42  p=0.16 | t=-0.59  p=0.56 | t=-0.03  p=0.98 | t=0.91  p=0.37 |
| Lifetime PTSD (CAPS) | t=0.48  p=0.63 | t=-1.22  p=0.23 | t=-1.78  p=0.08 | t=-1.39  p=0.17 | t=1.49  p=0.14 | t=0.71  p=0.48 |
| PSS - Avoidance | t=1.28  p=0.20 | t=0.45  p=0.65 | t=-1.06  p=0.29 | t=0.25  p=0.80 | t=0.81  p=0.42 | t=-0.91  p=0.36 |
| PSS - Intrusive | t=1.44  p=0.15 | t=-0.83  p=0.41 | t=-1.13  p=0.26 | t=-0.91  p=0.36 | t=-0.02  p=0.99 | t=0.21  p=0.84 |
| PSS - Hyperarousal | t=0.16  p=0.87 | t=1.38  p=0.17 | **t=-2.32**  **p=0.02** | t=0.55  p=0.58 | t=0.36  p=0.72 | t=-0.64  p=0.53 |
| Childhood Abuse - Physical | t=0.85  p=0.40 | **t=-2.36**  **p=0.02** | t=-0.74  p=0.46 | t=-0.10  p=0.92 | t=0.02  p=0.99 | t=0.98  p=0.33 |
| Childhood Abuse - Sexual | **t=3.27**  **p=0.001** | t=-1.17  p=0.25 | t=1.49  p=0.14 | t=-0.10  p=0.92 | t=1.03  p=0.31 | t=-1.33  p=0.19 |
| Childhood Abuse - Emotional | t=0.28  p=0.78 | **t=-2.64**  **p=0.01** | t=-0.60  p=0.55 | t=-0.31  p=0.76 | t=1.73  p=0.09 | t=0.98  p=0.33 |
| TEI score | t=-0.55  p=0.59 | t=0.51  p=0.61 | t=-1.64  p=0.10 | t=0.56  p=0.58 | t=-1.14  p=0.26 | t=0.44  p=0.66 |
| BDI Score | t=1.36  p=0.18 | t=0.74  p=0.46 | t=-0.46  p=0.65 | t=0.53  p=0.60 | **t=2.28**  **p=0.03** | t=-1.61  p=0.11 |

Statistically significant (**bold**) associations between stress-related metrics and leukocyte proportions, adjusted for age.

**NOTE:** **SUPPLEMENTAL TABLES XII-XIV** are embedded within **SUPPLEMENTAL FIGURES 8-9.**

**SUPPLEMENTAL FIGURES**


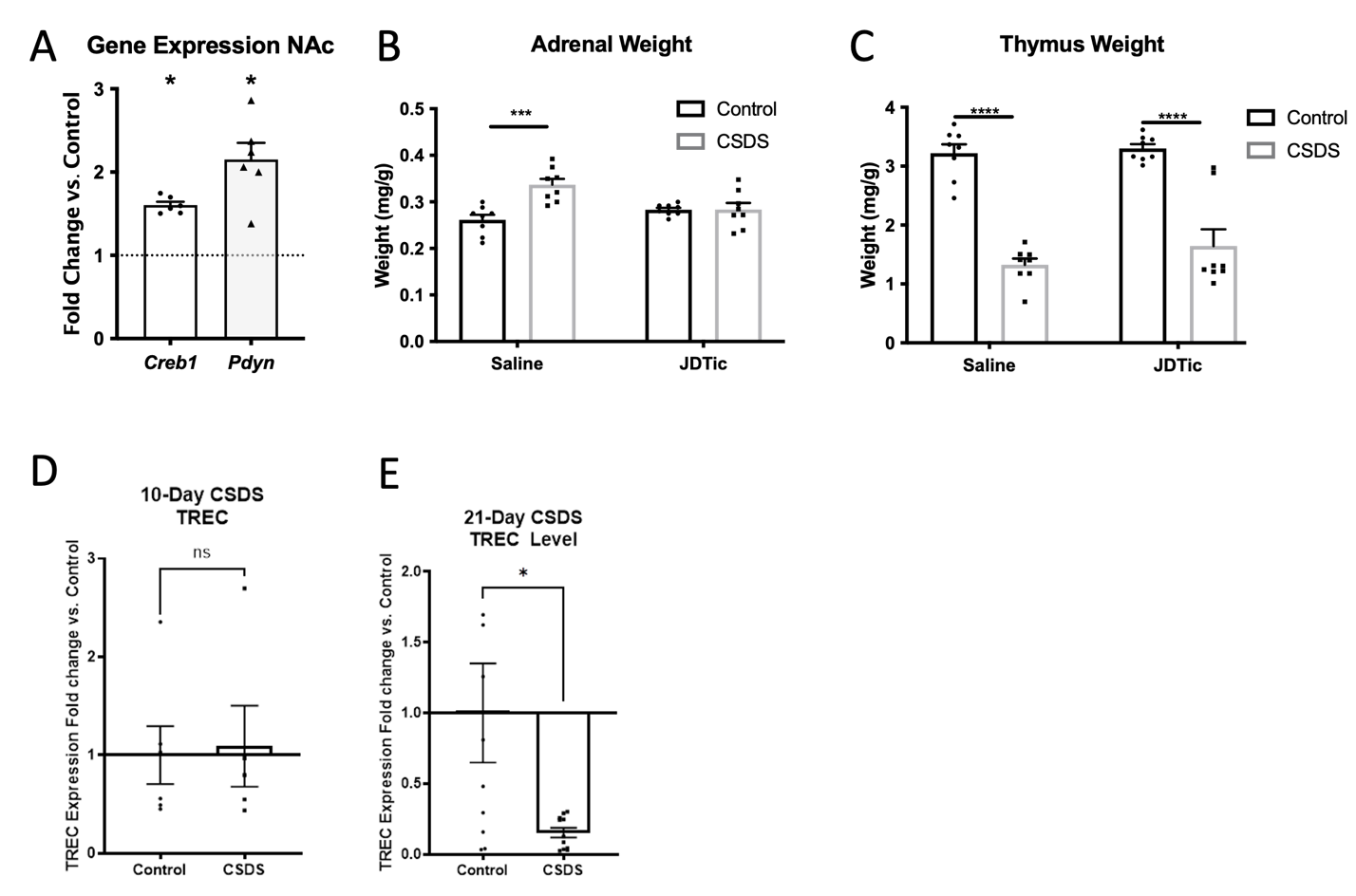


**S-Fig. 1** Physiological effects of Chronic Social Defeat Stress (CSDS). (**A**) A 10-day CSDS regimen caused increases in *Creb1* and *Pdyn* mRNA in the nucleus accumbens (NAc), reflecting activation of the brain kappa-opioid (KOR) systems. (**B**) The 10-day CSDS regimen also caused increases in adrenal gland weight that were blocked by the KOR antagonist JDTic, as well as (**C**) Reductions in thymus gland weight that were not affected by JDTic. (**D-E**) Reductions in TREC levels were detectable following the 21-day but not the 10-day CSDS regimen when changes are expressed as fold-change versus controls, replicating the pattern of results seen when expressed as changes in cycle thresholds (dCT). **p*<0.05 unpaired t-test and Mann-Whitney test (S1E), ****p*<0.001 Sidak’s Multiple Comparisons, N’s= 6-11/group.

**S-Fig. 2** Associations between TREC levels and age and childhood trauma in women. Note that TREC data are depicted as -dCT to clarify that larger dCT integer values indicate lower levels of TRECs. (**A**) TREC dCT values were normally distributed in women. (**B**) TREC levels were negatively correlated with age, and lower in women that experienced (**C**) childhood physical abuse or (**D**) childhood emotional abuse. TREC levels were also negatively correlated with (**E**) Becks Depression Inventory and (**F**) Modified PTSD Symptomatic Scale (mPSS) Hyperarousal score. ****p*<0.001, N=176.

**S-Fig. 3** Associations between TREC levels and age and childhood trauma in men. Note that TREC data are depicted as -dCT to clarify that larger dCT integer values indicate lower levels of TRECs. (**A**) TREC dCT values were normally distributed in men and (**B**) negatively correlated with age. N=122.

**S-Fig. 4** Associations between TREC levels and measures of leukocyte cell composition in women. Note that TREC data are depicted as -dCT to clarify that higher dCT integer values indicate lower levels of TRECs. Levels of TRECs were positively correlated with proportions of (**A**) CD8T cells, (**B**) CD4T cells, and (**D**) negatively correlated with proportions of neutrophils. In contrast, TREC levels were not correlated with proportions of (**C**) B-cells, (**E**) NK cells, or (**F**) monocytes. N=159

**S-Fig. 5** Associations between TREC levels and measures of leukocyte cell composition in men. Note that TREC data are depicted as -dCT to clarify that higher dCT integer values indicate lower levels of TRECs. Levels of TRECs were positively correlated with proportions of (**A**) CD8T cells, (**B**) CD4T cells, and (**C**) B-cells, and (**D**) negatively correlated with proportions of neutrophils. In contrast, TREC levels were not correlated with proportions of (**E**) NK cells or (**F**) monocytes. N=117

**S-Fig. 6** Associations of TREC with measures of epigenetic age acceleration in women. Note that TREC data are depicted as -dCT to clarify that higher dCT integer values indicate lower levels of TRECs. Levels of TRECs were negatively correlated with (**A**) HannumAge acceleration, (**B**) PheonAge acceleration and (**C**) extrinsic epigenetic age acceleration (EEEA). In contrast, TREC levels were not correlated with (**D**) DNAmAge acceleration, (**E**) GrimAge acceleration, or (**F**) intrinsic epigenetic age acceleration (IEAA). N=159

**S-Fig. 7** Associations of TREC with measures of epigenetic age acceleration in men. Note that TREC data are depicted as -dCT to clarify that higher dCT integer values indicate lower levels of TRECs. Levels of TRECs were negatively correlated with (**A**) HannumAge acceleration, (**B**) PhenoAge acceleration, (**C**) extrinsic epigenetic age acceleration (EEEA), and (**E**) GrimAge acceleration. In contrast, TREC levels were not correlated with (**D**) DNAmAge acceleration, or (**F**) intrinsic epigenetic age acceleration (IEAA). N=117


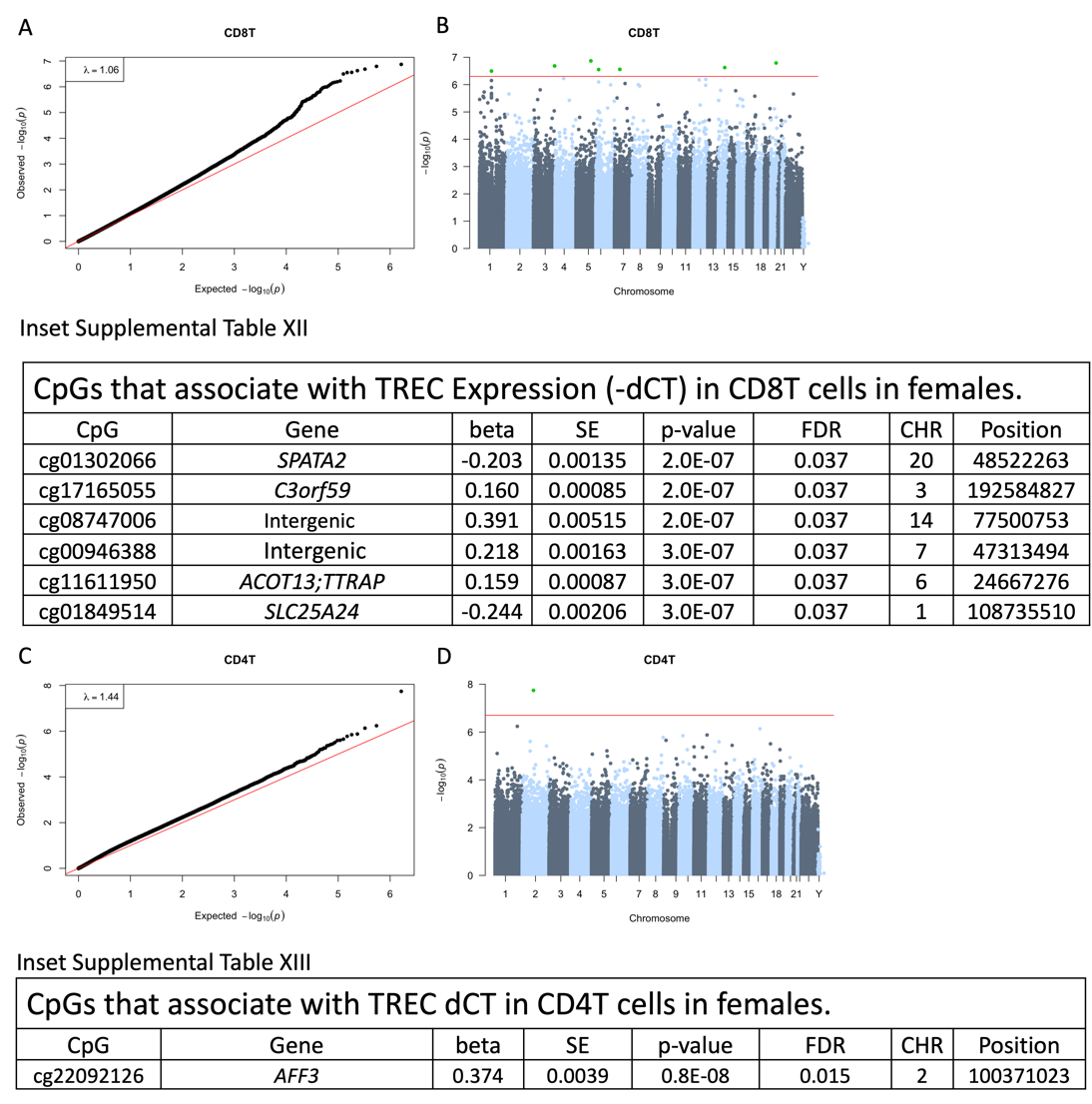


**S-Fig. 8** Associations of TREC levels with cell-type specific epigenome-wide association analysis (EWAS) in CD4T and CD8T cells in women. **(A)** QQ plots of CD8T cell specific EWAS for the comparison of 159 samples for which both TREC expression levels and methylation data were available. **(B)** Manhattan plot of CD8T cell specific EWAS of TREC levels. The y-axis is the –log10 of the unadjusted *p*-value for the association with TREC expression level. The red line indicates genome-wide EWAS statistical significance at FDR<0.05; **Inset Supplemental Table XII**. CpGs that associate with TREC expression levels in CD8T cells in women. The model is adjusted for age, sex, and smoking score; Beta: regression beta estimate; SE: standard error; FDR: false discovery rate; CHR: chromosome. **(C)** QQ plots of CD4T cell specific EWAS for the comparison of 276 samples for which both TREC expression level and methylation data were available. **(D)** Manhattan plot of CD4T cell specific EWAS of TREC level. The y-axis is the –log10 of the unadjusted p-value for the association with TREC expression level. The red line indicates genome-wide EWAS statistical significance at FDR<0.05; **Inset** **Supplemental Table XIII**. CpGs that associate with TREC expression levels in CD4T cells in women. The model is adjusted for age, sex, and smoking score; Beta: regression beta estimate; SE: standard error; FDR: false discovery rate; CHR: chromosome.


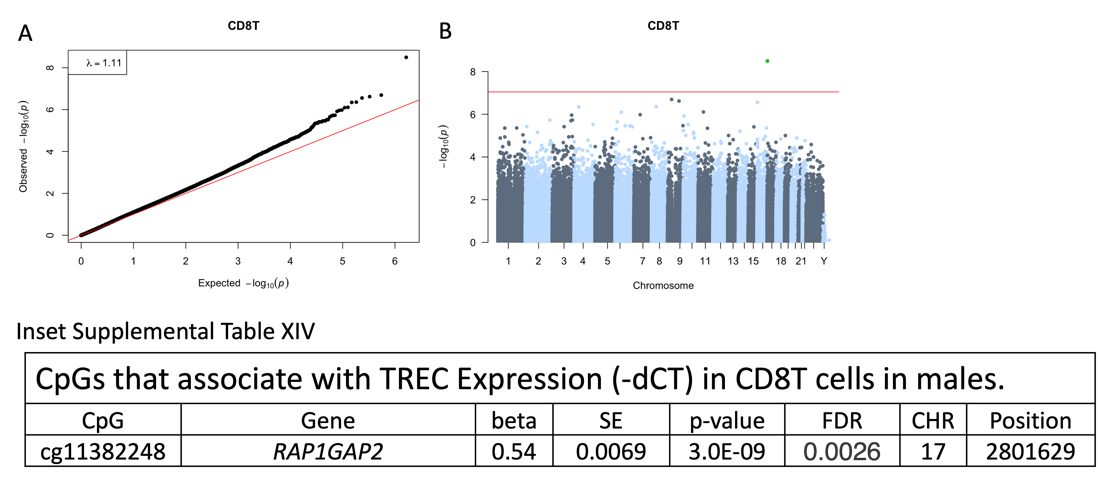


**S-Fig. 9** Associations of TREC levels with cell-type specific epigenome-wide association analysis (EWAS) in CD4T and CD8T cells in men. **(A)** QQ plots of CD8T cell specific EWAS for the comparison of 117 samples for which both TREC expression levels and methylation data were available. **(B)** Manhattan plot of CD8T cell specific EWAS of TREC levels. The y-axis is the –log10 of the unadjusted *p*-value for the association with TREC expression level. The red line indicates genome-wide EWAS statistical significance at FDR<0.05; **Inset Supplemental Table XIV**. CpGs that associate with TREC expression levels in CD8T cells in men. The model is adjusted for age, sex, and smoking score; Beta: regression beta estimate; SE: standard error; FDR: false discovery rate; CHR: chromosome.
